# Transactions of the Medical Association of Georgia—Remarks on the Pathology of Phlegmasia Dolens

**Published:** 1859-07

**Authors:** Hayden Coe

**Affiliations:** Atlanta, Georgia


### ARTICLE II.

*Transactions of the Medical Association of Georgia—Remarks on the Pathology of Phlegmasia Dolens.* By Hayden Coe, M. D. Atlanta, Georgia.

There is no disease in regard to which the profession have been more divided, than they have been as to the true nature, and Pathology of Phlegmasia Dolens. It has been located in almost every tissue, of which the affected limb is composed, by as many different authors. Besides, at one time it was thought to be a metastasis of the milk, the absurdity of which is very manifest. The other theories have all had their advocates, who have defended and sustained each his own theory with more or less ability, and not without some degree of plausibility.

But all have been laid aside as unsatisfactory to the profession, except one which was brought forward by Dr. Davis and afterwards supported by Dr. Lee, and others, (I mean Phlebitis) which is no more satisfactory than those which have preceded it. It will be wholly unnecessary for me to notice any of the theories which have been advanced, except the latter, (Phlebitis) they are too numerous and too well known to the profession. Dr. Churchill, after enumerating a half dozen theories or more, seems to think, that crural Phlebitis perhaps, is more satisfactory than any other, but says, "at the same time, it is not impossible that some further information may be necessary, before we fully comprehend the true theory of the disease." That crural Phlebitis may occur in some few instances of the many cases of Phlegmasia Dolens, we shall not attempt to deny, especially in those few cases which prove fatal; nor do we doubt in the least, that in those cases which Dr. Davis and Dr. Lee examined after death, that they found the veins inflamed and plugged up as they stated, and that this was most likely the real cause of death in those instances. But that Phlebitis does occur in all cases of Phlegmasia Dolens, or even in a majority of cases, and constitutes the Phenomena which occur in Phlegmasia Dolens, we do most positively *deny*; for we believe it to be impossible for any one to reconcile the symptoms which occur in this disease, or the treatment which is found to be most successful in the cure of it, with the theory of crural Phlebitis.

The distribution of the veins are unfavorable to this theory; Dr. Lee says, "that he has succeeded in tracing the inflammation of the Uterine veins, and that in most cases if not in all, the inflammation commences inthe uterine veins, and spreads along those veins to the illiac and then to the femoral vein." The uterine veins terminate in the internal illiac; if the inflamation commences in the uterine veins, why does not the inflamation spread along the internal illiac, and then the primitive illiac, and then the vena cava? why, after the inflammation reaches the primitive illiac, does the inflammation turn down the external illiac, to reach the femoral vein? Authors tell us that in Phlebitis, when the inflammation spreads, it is in the direction which the current of blood flows, that is, towards the heart.

Again, if it is Phlebitis, why is it that frequently just so soon as the disease in the limb first attacked begins to subside, the other limb is similarly attacked, and runs through a similar course?

Next, let us examine the symptoms as they usually occur in this disease.

Dr. Meigs says, (whch is high authority,) "Now gentlemen, see a limb supplied by a great femoral Artery and profunda, with its anterior and posterior tibials, and all the other branches; imagine, says he, these arteries healthy, strong, perfect in structure and volume, and see a current of blood driven into them by the contraction of the heart under the powerful excitement of a great fever, and then imagine the femoral vein or saphena obstructed by thickening of its endangium, by quantities of plastic lymph exuded from the inner wall, by a strong coagula of blood; for says he, the vein which carries the blood from the limb is as virtually compressed, as if you had tied it up with a ligature preparatory to bleeding; the vein is as if you had put the pad of Petit's tourniquet upon it, and left the artery perfectly free and unobstructed."

He says, "tell me what is to become of all the blood thrust into the limbs by the injecting force of an infuriated systemic ventricle, and how is it to get out?" *Sure enough* how is it to get out, if the veins are in the condition above described? And I ask, would not the consequences be of a much more serious character than usually occur in a case of Phlegmasia Dolens? Are the symptoms which occur in this disease, such as to warrant us in coming to any such conclusion, as that the veins are completely plugged up? Certainly not.

Says Dr. Meigs, "how is the woman to avoid the great swelling of the limbs?" I ask, is the swelling owing to Hyperemia? are the symptoms such as we would expect in Hyperemia?

If the veins are as effectually plugged up, as Dr. Meigs supposes, we must iuevitably have as the result, Hyperemia, and great swelling as a consequence. But, were we to apply a ligature around the limbs for bleeding, or the pad of a tourniquet to a vein, and obstruct the return of the blood through the vein, we would have swelling, it is true, but the limbs would be of a dark livid color, showing at once an excess of venous blood, attended with but little pain, amounting to nothing more than uneasiness. Whereas, in Phlegmasia Dolens, the swelling is attended with most acute pain, so much so, that at times it is almost beyond endurance, and with extreme sensibility, while, instead cf the limb being dark or livid, showing an excess of venous blood, the limb is even whiter than natural. It certainly is self evident that the obstruction of large veins must result in very serious consequences; unless soon removed, even if there was no disease of the veins at all, much more so than usually occurs in cases of Phlegmasia Dolens.

Again, says Dr. Meigs, "The inflammation of the endangium of the crural vessels dips through the fibrous coat, and causes infiltration into the common sheath, producing pain, essentially a neuralgia, by pressure on the crural nerve contained in the sheath with the crural vessels." But how can infiltration into the sheath produce pressure on the crural nerve, when only a very small branch of the crural nerve enters the sheath, or is connected with it in any way? The long saphenous nerve, enters the sheath it is true, but it is distributed mostly to the foot. Does this not entirely fail in accounting for the excruciating pain which occurs in Phlegmasia Dolens? Besides, usually the first and most acute pain is felt in the calf of the leg, and no portion of the crural nerve is distributed to that part of the leg. As I have said, most usually, but not always, the first symptom of Phlegmasia Dolens, is an acute pain in the calf of the leg. But from my own observation, I am inclined to think, that the pain in the calf of the leg is generally preceded by a dull pain, or uneasiness in the lumbar and iliac region, and about the hip, with tenderness or pressure in the region of the uterus; but it is of such a character, as not to have attracted any particular attenion from the patient, and after the attack of acute pain in the calf of the leg, it is entirely overlooked or forgotten by the patient, unless attention is especially directed to that fact. The acute pain is also evidently preceded by a restlessness, and an irritable condition of the whole nervous system, attended with more or less fever, not unfrequently of a remittent character. Again, what is the peculiar character of the great muscles of the calf of the leg, when attacked with this pain, which is very acute? The whole mass of muscle feels hard, as if firmly contracted, it feels as if firmly impacted against the bone, if you take hold of it and attempt to move it, you can not, and Dr. Meigs, makes this as one of his diagnostic symptoms of this disease. How is it that crural Phlebitis can so affect these particular muscles? Is it possible to reconcile the condition in which these muscles are found in this disease, with the theory of crural Phlebitis? We think not, we believe it to be utterly impossible.

Again, how is it that crural Phlebitis can render every part of the limb so exquisitely sensitive, so much so that the slightest touch produces severe pain, why is it that the pain and sensibility is not confined more particularly to the region of the Crural vessels? and why do we not see redness in the shin along the course of these vessels? (they are large and superficial) if the inflammation is confined to them

Again, it is said by some, that the fluid is serum, and is therefore the result of venous obstruction. If the fluid is serum why does not swelling commence in the foot? and gradually ascend up as in other cases of edematous swelling? In Phlegmasia Dolens the swelling sometimes commences in the thigh, sometimes in the calf of the leg, extending all over the limb in a few hours. It is evident then, that in the active stage of the disease, the fluid is not pure serum, but lymph, the hardness of the limb, and its failing to pit on pressure, prove that it is lymph. Besides, some authors tell us that it fails to be discharged, when incisions are made through the skin, therefore this fiuid cannot be the result of venous obstruction, but of excitement, as we shall attempt to show in the conclusion. Not unfrequently the treatment of disease assists us in understanding the nature of disease treated.—What is the treatment adopted, and found to be most successful in the treatment of this disease? is it not that given by Dr. Meigs? He says, "Take an old flannel petticoat, for where you find a woman you will find a petticoat, and wring it out of hot vinegar and wrap the whole limb up in it, and repeat it every few minutes for five or six hours, and then bath the limbs well with volatile linament with laudanum in it, and go on alternating the stuping and bathing the limb, until you relieve your patient." Some use poultices for a similar purpose, that he uses the stuping, I ask, would such a course of treatment as that above stated, be such as would be best calculated to relieve our inflammation of the serous coat of large veins? Would it subdue the imflammation in the vein? Is not such treatment much better calculated to relieve nervous irritation, than subdue inflammation? Just what is required in the treatment of Phlegmasia Dolens, if our notions of the nature of the disease are correct, so then, the distribution of the veins, the manner in which both limbs are affected, the symptoms both general and local, the treatment of the disease, the fatality of the disease, perhaps not one in a hundred, if one in five hundred, proving fatal, all are in direct opposition to the theory of Phlebitis,

No doubt but inquiry has already been instituted in the minds of those who hear me, that, if it is not Crural Phlebitis, what is it, what is the nature of this disease? What its true Pathology.

We believe it to be a reflex disease through the medium of the nervous system, which, has its primary seat in lying in women, in the uterus. The intimate connection between the spine and uterus is manifest no doubt to all of you, from observation, that is, if any irritation is applied to the uterus, as pressure on the os, or disease of the os it produces more or less pain in the back. The condition of the lying in woman is such as to be very favorable to the development of this disease at this time, owing to the irritability of the nervous system, which is so manifest at this period. But that this disease is confined to the lying in woman, or even to females, or to the inferior extremities, we do not believe. Nor do we believe, that it is confined to irritation primarily situated in the Uterus. We believe that a similar disease, if not identically the same, may arise from irritation of the bowels, long and constantly continued, also from cancer of the breast, as we have some such cases reported.

It may be necessary before we proceed further, to say a few words in reference to reflex action, about which there has been so much said of late, and I trust not without benefit to the profession.

1st. Then, the spinal column is not a mere bundle of nerve fibres for conveying impressions made upon the periphery of the sensitive nerves to the brain, and impressions from the brain through the motor nerves, as was once supposed, but is composed of seperate centres, each pair of spinal nerves having their own centre connected by commissures, through which impressions made upon the periphery of the nerves, are transmitted to the brain.

Impressions made on the sympathetic nerve, not only reach the ganglia of that nerve, but are also conveyed from the ganglia situated on each side of the vertebra to these spinal centres, and through them to the brain, precisely as the impressions made on the periphery of the sensitive nerves are conveyed. Reflex action can be effected through these centres in the spinal cord, as well as through the ganglia of the sympathetic nerve. Every tissue has its own peculiar irritability, which is capable of being acted upon by proper stimuli, this stimuli must be applied, in one of two ways, either by the direct application of the stimulus to the tissue itself, or through the medium of the nervous system. Sensibility is not irritability, they are seperate and distinct properties, sensibility belongs to the nervous system alone, but may act as a stimulus to irritability.

If we apply an irritant directly to the fibres of a muscle, it acts on the irritability of the mucular fibre, exciting mobility.

Again, If we apply our irritant to the root of the motor nerve, which supplies the muscle, it acts on the irritability of the muscle, exciting mobility in the muscular fibres, precisely as if the irritant had been applied directly to the muscular fibre itself. Again, if we apply our irritant to the periphery of a sensitive nerve, it so acts on the root of the motor nerve, through the nervous Centre in the spinal column, as to cause the motor nerve, to act on the irritability of the muscular fibre, exciting mobility in the muscle, producing what we term sensori motor action.

Again, if an impression is made by an irritant upon the sympathetic nerve, it may be conveyed to the ganglia of that nerve, and from these ganglia or ganglionic nervous centers, conveyed to the spinal centers, and acts through these spinal centers upon the roots of the motor nerves, precisely as if the impression had been made upon the periphery of the sensitive spinal nerves, producing the same sensori motor action in the muscle. Again, if we irritate the root of a sensitive nerve, or any portion of the nerve between the root and the periphery of the nerve, (the root being connected with the spinal center), we have at once a disturbance ot sensibility, which acts upon the irritibility of the tissue it supplies, precisely as if the irritant had been applied directly to the tissue itself. Lastly, an irritant applied to the periphery of a sensitive nerve, or to the sympathetic nerve, may, through the spinal center, not only act on the roots of the motor nerves, producing sensori motor action, but so affect all the roots of the sensitive nerves arising from the same centers, as if we were to apply the irritant directly to each sensitive root itself; consequently, increasing the sensibility, or otherwise disturbing the sensibility of these nerves, which act upon the irritability of the tissues supplied by these nerves, precisely as if the irritant had been applied to the tissue itself. Nerve fibres which supply a tissue, if they pass through a plexus, as most of them do, may have their origin in a half dozen centers or more.

We have said, that each tissue has its own peculiar irritability, which was capable of being acted on either by stimuli being applied directly to the tissue itself, or through the medium ot the nervous system, as we have endeavored to describe. B[illegible] illustrate, if we apply an irritant to muscular fibre, it contracts, but if you apply an irritant to a serous membrane, a grain of sand for instance, we have an increase of its function, which is secretion, with increased sensibility, if applied to the capillaries, they like the muscles contract, if to the mucous membrane, or glandular tissue, we have also increased secretion as the result.

And any agent acting on the sensitive nerves, either directly or indirectly, affecting their sensibility, produces precisely the same affect upon the irritability of the tissues, which they supply, as if the agent had been applied directly to the tissues themselves.

I have been thus tedious in order to establish the fact, that an irritant applied to the periphery of a sensitive nerve, or to the sympathetic nerve, was capable of affecting the spinal center from which it arose, or was connected, so as to affect all the roots of the sensitive nerves, arising from the same centre, precisely, as if the irritant had been applied directly to the root itself, and where a nerve passes through a plexus, it may have its origin in a half dozen centers, as is the case with the lumbar and sacral nerves. But to return to our disease, Phlegmasia Dolens—we said that the primary seat of the disease was situated in the uterus. The uterine nerves are derived from the aortic and hypogastric plexus, with some branches directly from the sacral plexus, an irritant applied to the periphery of the uterine nerves, produces an impression, which is conveyed to the hypogastric plexus, from thence to the lumbar ganglia situated on the sides of the bodies of the vertebra, and from these ganglia to the spinal centres in the lumbar portion of the spinal column, and impressions are conveyed in a similar manner, to the nervous centers in the sacrum, or more directly, by impressions on the peripheral branches of the sacral nerves distributed to the uterus, affecting these centers, so as to produce the same affect on the roots of the sensitive nerves, as if the irritant had been applied to the root itself, and as a result, the sensibility of these nerves, arising from these centres, is greatly increased, and now acts as a stimulus to the irritability of every tissue to which they are distributed. Just precisely what occurs in Phlegmasia Dolens.

We can now account for the extreme sensibility of the whole limb, and why every tissue of which the limb is composed, seems to be disordered. The nerves arising from these spinal centers are distributed to every tissue of which the limb is composed. The roots of the sensitive nerves being affected in the same manner, as if the irritant had been applied to the root itself, the sensibility is greatly increased, and this extreme sensibility acts as a stimulus on the irritability of each tissue of which the limb is composed, precisely as if an irritant had been applied directly to each tissue, producing precisely the same result, that is, acting on the muscles, causing them to contract, on the capillaries causing them to contract, on the areolar tissue, causing an increased secretion, producing an effusion as the result, and even the coats of the veins and lymphatics may be in an irritable condition. This is precisely what would occur, if an irritant had been applied directly to each one of these tissues, and is the condition in which we find the tissues of the limb, affected with Phlegmasia Dolens. We can now account for the exquisite sensibility of the whole limb. We can account for the acute pain in the calf of the leg, and why the muscles feel so hard, as if impacted against the bone, as if in a state of tonic contraction, which they are, so much so, that you can not move them. There are 4 or 5 large muscular branches of nerves distributed to these muscles, in this injured condition of their sensibility, which acts as a powerful stimulus, on the irritability of these muscles, putting them in this painful and contracted condition, which we find. Sometimes we find the large muscles about the hip and thigh in a simular condition.

We can also explain why the limb looks whiter than natural, as we know it does in this disease. The increased sensibility of the nerves acts on the irritability of the capillaries, causing them to contract, preventing the usual amount of red globules from entering these vessels. We can also account for the nature of the fluid—it is not common serum; it is an exhalation from the excitant in the areolar tissues, as we know is the result, when an irritant is applied to a fibrous membrane, this is the reason why it is lymph instead of serum, as we know it is by the limb failing to pit only in the last stage of the disease, after the excitement has ceased. Another proof of its being lymph, is, the swelling seldom, if ever, commences in the foot.

The swelling is produced by the contraction of the muscles, and the effusion.

We can readily account for the cord, which is sometimes found in the region of the crural veins, and so much relied on by the supporters of Phlebitis. The vessels are surrounded by an abundance of loose areolar tissue, besides, they are enclosed in a fibrous sheath, these like another fibrous tissue when irritated, throw out lymph, producing infiltration of this fluid in these tissues, producing the cord.

We can very readily see why in a few instances, perhaps, all that prove fatal, the inflammation might extend from the sheath to the fibrous coat of the vein, and from the fibrous coat to the serous coat, and as the result, prove fatal, for we think the inflammation commencing in the sheath, could as easily extend to the serous membrane, as for inflammation commencing in the serous membrane to extend to the sheath. We can also account for the inability to move the limb, which occurs in this disease, even after the sensibility has subsided.

We can account with more plausibility, for the manner in which both are sometimes affected, by the irritation extending from one portion of the nervous center to the other through its connecting Commissure. Another proof of our, theory is, its results, it seldom ever proves fatal; perhaps, never,unless the inflammation extends from the sheath of the vessels to the serous membrane. The treatment of this disease, is a farther proof of our theory of the pathology of this disease, stuping the limb with flannel wrung out of hot vinegar, enveloping the whole limb, is much better calculated to relieve increased sensibility and irritability of the tissue, than sudden Phlebitis; as we see it does do, when effectually applied; also the bathing the limb in volatile Lineament and laudanum, is another remedy, that would relieve irritation, and there is some reason for such a course of treatment, as is adopted in the treatment of this disease, with the view we take of its Pathology. But if it is Phlebitis we can offer no reason in the world, why such a course of treatment should be successful or even beneficial. One important point in the treatment of this disease is to allay the irritation in the Uterus, by using mucilaginous washes with laudanum. I have also used quinine combined with an opiate with decided benefit in this disease. A similar disease may occur in the superior extremity, from cancer or disease of the breast, by an impression made on the periphery of the thoracic nerves, which is conveyed to the nervous centres in the spinal chord in the cervical region, also a similar disease has been produced in protracted cases of typhoid fever, the primary seat of which, no doubt was located in the mucous membrane of the bowels, several cases are refered to by Dr. Tweedie, the explanation of which, would be similar to the one I have already given and as I have been much more tedious in my explanation than I anticipated, I will close, hoping this subject may be more thoroughly investigated.

